# Atorvastatin Attenuates Diet-Induced Non-Alcoholic Steatohepatitis in APOE*3-Leiden Mice by Reducing Hepatic Inflammation

**DOI:** 10.3390/ijms24097818

**Published:** 2023-04-25

**Authors:** José A. Inia, Geurt Stokman, Elsbet J. Pieterman, Martine C. Morrison, Aswin L. Menke, Lars Verschuren, Martien P. M. Caspers, Martin Giera, J. Wouter Jukema, Anita M. van den Hoek, Hans M. G. Princen

**Affiliations:** 1 Department of Metabolic Health Research, The Netherlands Organization for Applied Scientific Research (TNO), 2333 BE Leiden, The Netherlands; 2 Department of Cardiology, Leiden University Medical Center (LUMC), 2333 ZA Leiden, The Netherlands; 3 Einthoven Laboratory for Experimental Vascular Medicine, Leiden University Medical Center (LUMC), 2300 RC Leiden, The Netherlands; 4 Department of Microbiology and Systems Biology, The Netherlands Organization for Applied Scientific Research (TNO), 2333 BE Leiden, The Netherlands; 5 Center for Proteomics and Metabolomics, Leiden University Medical Center (LUMC), 2333 ZC Leiden, The Netherlands; 6 Netherlands Heart Institute, 3511 EP Utrecht, The Netherlands

**Keywords:** atorvastatin, NAFLD, NASH, inflammation, fibrosis, inflammasomes, gene expression

## Abstract

Patients with metabolic syndrome are often prescribed statins to prevent the development of cardiovascular disease. Conversely, data on their effects on non-alcoholic steatohepatitis (NASH) are lacking. We evaluated these effects by feeding APOE*3-Leiden mice a Western-type diet (WTD) with or without atorvastatin to induce NASH and hepatic fibrosis. Besides the well-known plasma cholesterol lowering (−30%) and anti-atherogenic effects (severe lesion size −48%), atorvastatin significantly reduced hepatic steatosis (−22%), the number of aggregated inflammatory cells in the liver (−80%) and hepatic fibrosis (−92%) compared to WTD-fed mice. Furthermore, atorvastatin-treated mice showed less immunohistochemically stained areas of inflammation markers. Atorvastatin prevented accumulation of free cholesterol in the form of cholesterol crystals (−78%). Cholesterol crystals are potent inducers of the NLRP3 inflammasome pathway and atorvastatin prevented its activation, which resulted in reduced expression of the pro-inflammatory cytokines interleukin (IL)-1β (−61%) and IL-18 (−26%). Transcriptome analysis confirmed strong reducing effects of atorvastatin on inflammatory mediators, including NLRP3, NFκB and TLR4. The present study demonstrates that atorvastatin reduces hepatic steatosis, inflammation and fibrosis and prevents cholesterol crystal formation, thereby precluding NLRP3 inflammasome activation. This may render atorvastatin treatment as an attractive approach to reduce NAFLD and prevent progression into NASH in dyslipidemic patients.

## 1. Introduction

Non-alcoholic fatty liver disease (NAFLD) is emerging worldwide as the most prevalent form of liver diseases and is considered the hepatic manifestation of the metabolic syndrome. The number of NAFLD patients is rising and therefore more patients progress to the chronic (systemic) inflammatory state of NAFLD, known as non-alcoholic steatohepatitis (NASH) [1].

Hyperlipidemia and systemic inflammation are well-known risk factors for NASH [2]. Patients exhibiting hyperlipidemia are often prescribed HMG-CoA reductase inhibitors, i.e., statins, as a first line of defense to prevent development and stall progression of cardiovascular disease as a result of elevated levels of low density lipoprotein (LDL) cholesterol and other apolipoprotein B-containing lipoproteins. The anti-inflammatory effects of statins with respect to cardiovascular disease have been described in both humans [3,4] and mice [5,6] and can be attributed to different mechanisms, such as through reduction of NFκB pathway activation [7]. In the context of clinical NAFLD-NASH, conflicting data have been reported on the effects of statins, though recent studies advocate their beneficial effects on hepatitis and liver fibrosis [8,9]. Animal studies can therefore be most helpful in providing more insight into whether statins can affect NASH development and, if so, by what mechanisms.

Concerns have been raised about whether currently available preclinical NAFLD-NASH models accurately mimic the disease, given that the used experimental conditions may be less relevant to the clinical setting. These models often accentuate specific features of NAFLD-NASH and are therefore highly useful for investigation of pathogenic processes. Nevertheless, NAFLD-NASH models are often not suitable for evaluation of lipid-lowering interventions, since most models do not respond well to statins and other lipid-lowering drugs [10,11,12]. For the present study, we used mice expressing the human apolipoprotein E3-Leiden (APOE*3-Leiden) gene isoform, a translational hyperlipidemic mouse model with an intact ApoE-LDL-receptor-mediated clearance pathway [13]. This model closely reflects metabolic and histological features of human NASH and hepatic fibrosis while simultaneously developing atherosclerosis when fed a Westernized diet [12,14,15,16]. Most importantly, APOE*3-Leiden mice respond well to all lipid-lowering interventions that are used in the clinic including statins, ezetimibe, fibrates and PCSK9 and ANGPTL3 inhibitors [5,10,11,12,13,14,17,18,19]. Here, APOE*3-Leiden mice were treated with a statin dose that resulted in a decrease in total cholesterol levels of approximately 30%, a clinically relevant therapeutic effect size that is representative for the response to statins of a large group in the mildly hyperlipidemic patient population [20]. Using histological, biochemical, lipidomic and gene expression analyses, we demonstrated that atorvastatin has pronounced effects on different inflammatory aspects in this diet-induced NASH mouse model, and as such provides a rationale for use in NAFLD-NASH patients, either alone or on top of more specific NASH interventions.

## 2. Results

### 2.1. Metabolic Risk Factors and Atherosclerosis Parameters Are Improved by Atorvastatin

In the APOE*3-Leiden model, body weight at study endpoint was similar for all groups and caloric food intake was slightly lower in WTD-fed mice as compared to chow-fed mice (Table 1). Upon WTD feeding, APOE*3-Leiden mice developed hyperlipidemia as reflected by a 10.7-fold increase in plasma cholesterol levels and 97% increase in triglyceride levels. As expected, atorvastatin-treated mice had significantly lower plasma cholesterol (−43%) and triglyceride levels (−44%) as compared to the WTD control group. WTD feeding resulted as well in an increased liver weight (+91%) and a 5.3-fold and 6.9-fold increase in the liver function markers plasma ALT and AST, respectively, in comparison to healthy, chow-fed mice. Atorvastatin limited this increase in liver weight (−29%) and significantly reduced plasma ALT levels (−55%) and plasma AST levels (−33%) as compared to WTD-fed control mice. Plasma levels of the acute phase protein serum amyloid A (SAA) increased significantly in response to WTD feeding (+102%) and were significantly reduced by atorvastatin (−42%) to levels comparable to chow controls.

Total cholesterol exposure in atorvastatin-treated mice was significantly reduced with 30% (Figure S1A), resulting in a 28% reduction in the number of atherosclerotic lesions (Figure S1B,C). Moreover, atherosclerotic plaques from atorvastatin-treated mice had a higher stability index (+42%), calculated by dividing the sum of smooth muscle cell and collagen content as plaque stabilizing factors by the sum of macrophage and necrotic core content as plaque destabilizing factors (Figure S1D). While the area of mild (type I-III) atherosclerotic lesions did not differ between atorvastatin-treated mice and WTD-fed mice that did not receive intervention, there was a significant reduction in the lesion area of moderate (type IV) and severe (type V) atherosclerotic lesions in mice on the WTD supplemented with atorvastatin (−48%) (Figure S1E). These data confirm the beneficial effects of atorvastatin on atherosclerosis development in a NAFLD-NASH setting.

### 2.2. Atorvastatin Mitigates Hepatic Steatosis and Strongly Reduces Hepatic Inflammation and Fibrosis

**Figure 1:**
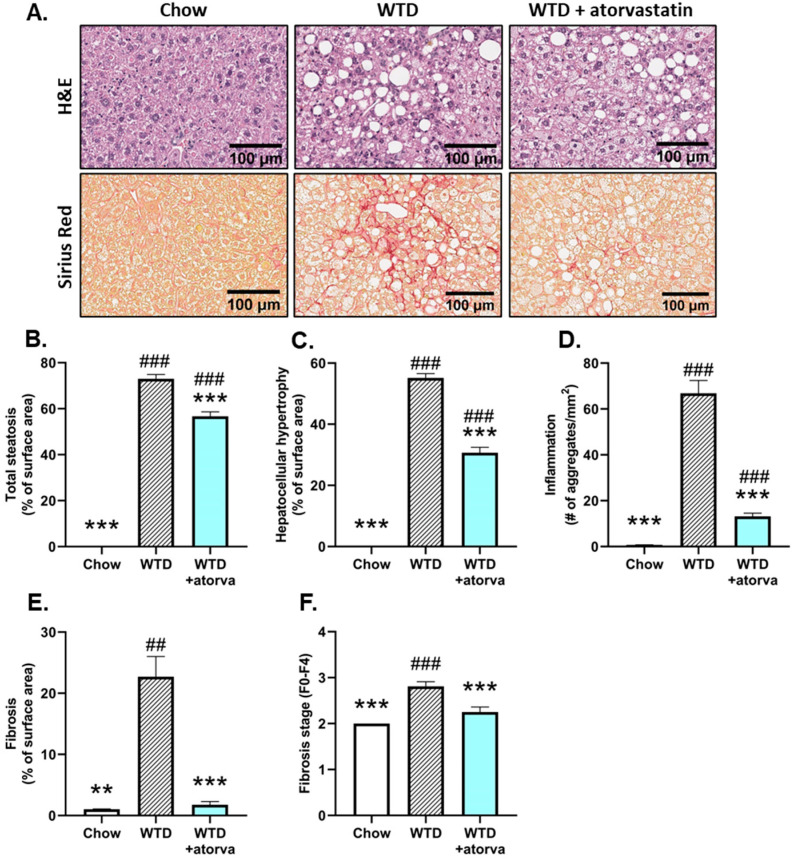
Histological photomicrographs and quantitative analysis of NASH parameters. APOE*3-Leiden mice were fed a Western-type diet (WTD) with (*n* = 16) or without (*n* = 16) atorvastatin admix for 32 weeks. Chow-fed mice (*n* = 10) were included as a healthy reference. Representative histological photomicrographs of H&E-stained and Sirius Red-stained liver cross-sections (**A**). Total steatosis (**B**) and hepatocellular hypertrophy (**C**) as percentage of surface area, number of inflammatory foci per mm^2^ microscopic field (**D**), fibrosis as percentage of surface area (**E**) and fibrosis stage (F0–F4) (**F**) were determined at the study endpoint (t = 32 weeks) and are presented as mean ± SEM. ** *p* < 0.01, *** *p* < 0.001 vs. WTD. ## *p* < 0.01, ### *p* < 0.001 vs. chow.

NASH development was histologically analyzed and WTD feeding resulted in pronounced hepatic steatosis, inflammation and fibrosis compared with the chow-fed animals (Figure 1A). Atorvastatin limited hepatic steatosis, as reflected by the lower percentage of surface area of total steatosis, based on histopathological scoring of the percentage of steatotic hepatocytes per total surface area (−22%) (Figure 1B). In association with hepatic steatosis, WTD-feeding induced hepatocellular hypertrophy that was significantly reduced in atorvastatin-treated mice (−44%) (Figure 1C). Furthermore, hepatic inflammation was significantly lower in atorvastatin-treated mice when compared to mice that received WTD without treatment, based on a robust decrease in the number of inflammatory cell aggregates (−80%) (Figure 1D). Sirius Red-stained liver tissue was scored for fibrosis and revealed that the WTD induced pronounced fibrosis, which was fully prevented by atorvastatin (−92%) (Figure 1E), resulting in an almost similar appearance as was observed in the chow-fed group (Figure 1A). Mice that received the WTD developed bridging fibrosis (stage F3), while in atorvastatin-treated mice, fibrosis was mostly observed in the perisinusoidal/perivenular and periportal area (stage F2) (Figure 1F).

### 2.3. Atorvastatin Decreases Hepatic Cholesterol Content, Strongly Reduces the Formation of Hepatic Cholesterol Crystals and Improves the Lipidomic Profile

**Figure 2:**
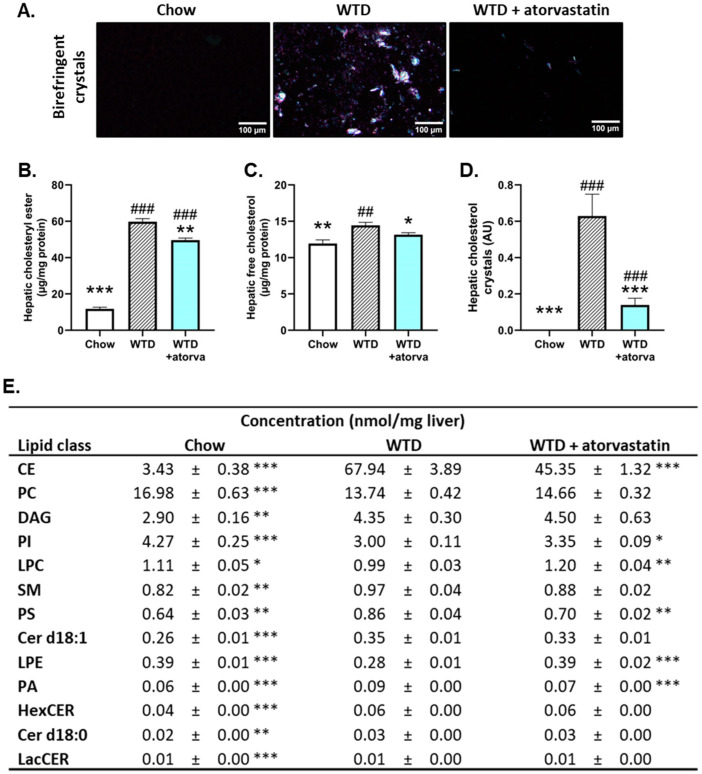
Atorvastatin decreases hepatic cholesterol content and strongly reduces formation of cholesterol crystals in the liver. APOE*3-Leiden mice were fed a Western-type diet (WTD) with (*n* = 16) or without (*n* = 16) atorvastatin admix for 32 weeks. Chow-fed mice (*n* = 10) were included as a healthy reference. Representative photomicrographs of liver cryosections showing birefringent cholesterol crystals (**A**). Hepatic cholesteryl ester (**B**), free cholesterol (**C**), cholesterol crystal content (**D**) and hepatic concentrations of lipid species that were significantly altered in WTD-fed mice compared to chow-fed mice (**E**) were determined at the study endpoint (t = 32 weeks) and are presented as mean ± SEM. * *p* < 0.05; ** *p* < 0.01; *** *p* < 0.001 vs. WTD; ## *p* < 0.01; ### *p* < 0.001 vs. chow. CE, cholesteryl ester; Cer, ceramide; DAG, diglyceride/diacylglycerol; HexCER, hexosylceramide; LacCer, lactosylceramide; LPC, lysophosphatidylcholine; LPE, lysophosphatidylethanolamine; PA, phosphatidic acid; PC, phosphatidylcholine; PI, phosphatidylinositol; PS, phosphatidylserine; SM, sphingomyelin.

The main mode of action of statins is inhibition of HMG-CoA reductase, resulting in impaired cholesterol synthesis. Biochemical and histological analysis of hepatic cholesterol was performed to provide insight into the effects of atorvastatin on hepatic cholesterol storage. WTD feeding induced a significant 4.0-fold increase in hepatic cholesteryl ester content compared to chow-fed mice, that was reduced (−17%) with atorvastatin (Figure 2B). Since intrahepatic cholesterol, either as free cholesterol or free cholesterol in the form of cholesterol crystals, is a very potent inducer of hepatic inflammation [21] and is elevated in human NASH as well [22], we determined the effect of atorvastatin on both parameters. Microscopic analysis of liver cross-sections under polarized light revealed that WTD-feeding caused pronounced formation of large birefringent crystals (Figure 2A). Atorvastatin prevented the rise in free cholesterol levels (−9%) (Figure 2C) and almost fully prevented the formation of hepatic cholesterol crystals (−78%) (Figure 2D).

Since high concentrations of certain lipid species have been linked with worsening of the metabolic syndrome and NASH, we sought to determine how the WTD affects the hepatic lipidomic profile in APOE*3-Leiden mice and how atorvastatin affects this profile. WTD feeding induced significant changes in the concentrations of thirteen lipid classes (Figure 2E). In particular, concentrations of hepatic cholesteryl esters were elevated in WTD-fed mice compared to chow-fed mice (18.8-fold increase), an elevation that was partially prevented in atorvastatin-treated mice (−33%). These data are in line with the previously shown biochemically determined data on cholesteryl esters (Figure 2B). Furthermore, significant changes in the lipid concentrations with atorvastatin intervention were also observed for phosphatidylinositol (PI), lysophosphatidylcholine (LPC), phosphatidylserine (PS), lysophosphatidylethanolamine (LPE) and phosphatidic acid (PA). In all these cases, lipid concentrations were more comparable with those observed in healthy, chow-fed mice. Together, these data show that the WTD has strong effects on many lipid species and that atorvastatin largely prevents the accumulation of lipid species in the liver.

### 2.4. Atorvastatin Reduces Immune Cell Infiltration and NLRP3 Inflammasome Pathway Activity

**Figure 3:**
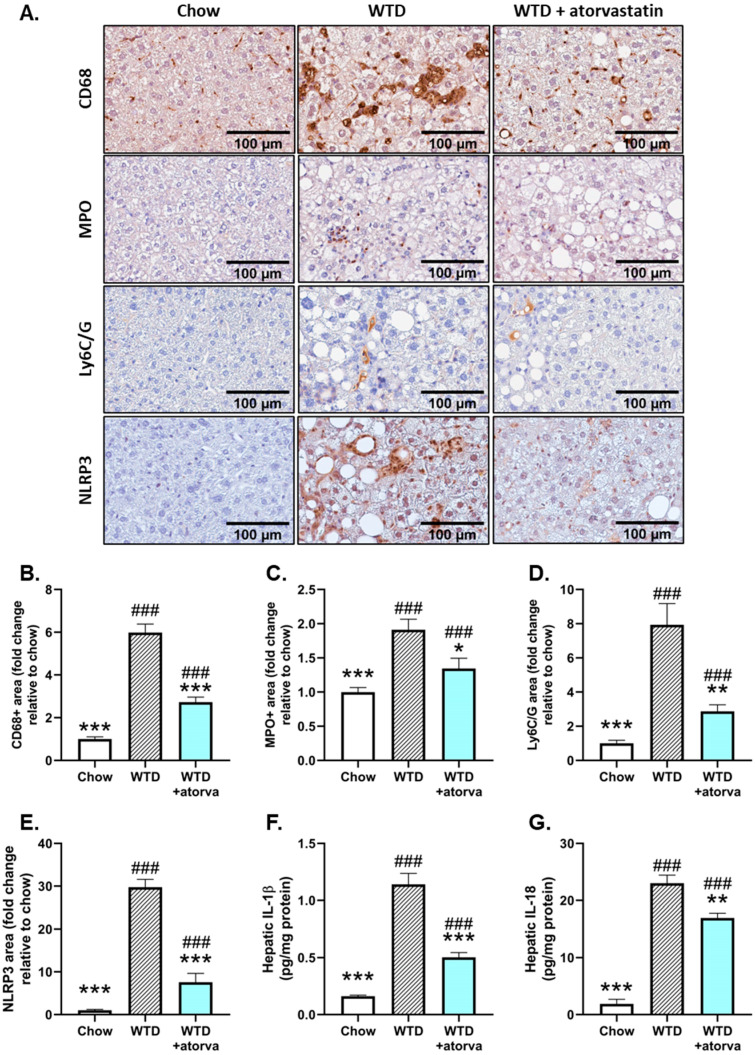
Atorvastatin ameliorates inflammatory cell infiltration and NLRP3 inflammasome activation in the liver. APOE*3-Leiden mice were fed a Western-type diet (WTD) with (*n* = 16) or without (*n* = 16) atorvastatin admix for 32 weeks. Chow-fed mice (*n* = 10) were included as a healthy reference. Histological photomicrographs of liver cross-sections stained for CD68, myeloperoxidase (MPO) Ly6C/G and NLRP3 (**A**). Liver CD68, MPO, Ly6C/G and NLRP3 content as measured by percent positive area and expressed as a fold-change relative to chow (**B**–**E**, respectively) and hepatic protein levels of IL-1β (**F**) and IL-18 (**G**) measured by ELISA at the study endpoint (t = 32 weeks). Data are presented as mean ± SEM. * *p* < 0.05, ** *p* < 0.01, *** *p* < 0.001 vs. WTD. ### *p* < 0.001 vs. chow.

Since atorvastatin strongly limited hepatic inflammation as measured by the number of inflammatory aggregates, we subsequently analyzed in more detail the nature of these inflammatory cells. For this purpose, we stained liver sections immunohistochemically for cluster-of-differentiation 68 (CD68), a marker of resident Kupffer cells and infiltrated macrophages and observed that in atorvastatin-treated mice, there was a significant reduction in CD68-positive cells when compared to WTD-fed mice (−54%) (Figure 3A,B). Similar reductions in positively stained area could be observed for myeloperoxidase (MPO), as marker of activated neutrophils (−25%) (Figure 3A,C) and Ly6C/G as marker for granulocytes (−64%) (Figure 3A,D).

To investigate the background of this decreased infiltration of innate immune cells and since previous studies have shown that cholesterol crystals are potent inducers of the NLRP3 inflammasome [21,23] and were shown here to be strongly reduced by atorvastatin, we investigated whether atorvastatin affected activation of the NLRP3 inflammasome pathway by measuring hepatic NLRP3 expression and expression of IL-1β and IL-18. Immunostaining of liver tissue for NLRP3 revealed a strong increase in the positively stained area in WTD-fed mice compared to chow-fed mice (Figure 3A,E) that induced in the downstream a 6.8-fold increase in hepatic IL-1β protein levels and 11.1-fold increase in hepatic IL-18 protein levels (Figure 3F,G). The reduced formation of hepatic cholesterol crystals in atorvastatin-treated mice prevented the rise in hepatic NLRP3 expression (Figure 3E). The consequent lowering of pro-inflammatory cytokine concentrations (−61% for IL-1β and −26% for IL-18) (Figure 3F,G) emphasizes the beneficial effects that atorvastatin exerts on hepatic inflammation and further supports its proposed effect on NLRP3 inflammasome activation.

### 2.5. The Transcriptome Profile of WTD-Fed Mice Is Ameliorated by Atorvastatin

**Figure 4:**
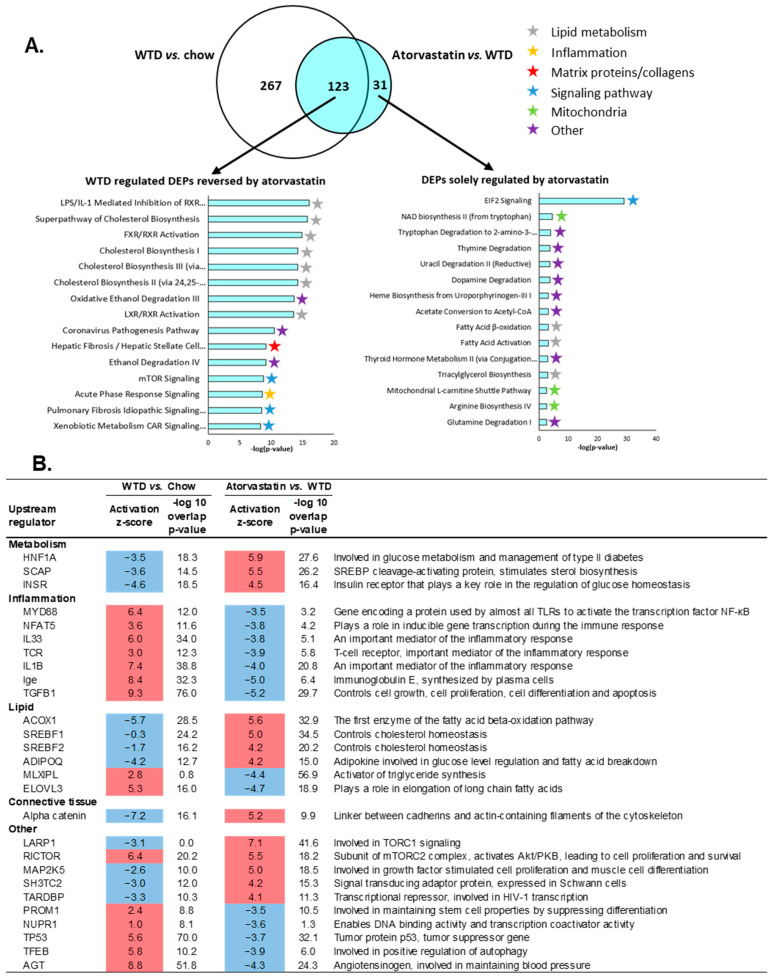

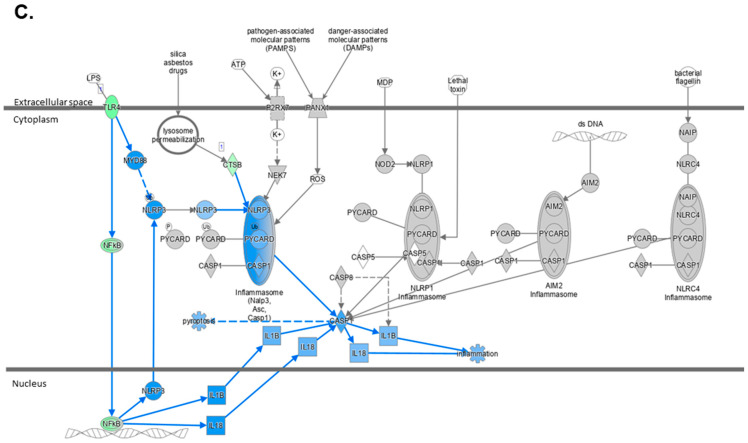
Atorvastatin improves the hepatic transcriptome profile of WTD-fed APOE*3-Leiden mice. APOE*3-Leiden mice were fed a Western-type diet (WTD) with (*n* = 16) or without (*n* = 16) atorvastatin admix for 32 weeks. Chow-fed mice (*n* = 10) were included as a healthy reference. (**A**) Venn diagram illustrating the overlap of differentially expressed pathways, with the white circle representing WTD vs. chow-fed mice and the blue circle indicating atorvastatin-treated mice vs. WTD without treatment. The top 15 most significantly enriched pathways for pathways reversed by atorvastatin and the top 15 pathways solely regulated by atorvastatin are shown. (**B**) Upstream regulator analysis showing the predicted activation state of transcription factors/master regulators, expressed as z-score. The overlap *p*-value indicates significant enrichment of genes downstream of an upstream regulator and a *p*-value < 0.01 was considered statistically significant. Upregulation is indicated with red color and downregulation with blue color. Ranking is based on z-scores from the largest upregulation to largest downregulation by atorvastatin by category. (**C**) Hepatic gene expression changes in mice on WTD supplemented with atorvastatin vs. WTD alone where green color indicates decreased measurement of RNA levels and blue color indicates model-predicted inhibition.

To further explore the anti-inflammatory effects of atorvastatin in the liver, transcriptome analysis was performed to identify pathways and upstream (master) regulators that were differentially expressed in statin-treated mice compared to untreated WTD-fed mice. The WTD induced differential expression of 390 pathways compared to APOE*3-Leiden mice on the healthy chow diet (Figure 4A, Venn diagram, white circle). Compared to untreated WTD mice, atorvastatin modulated 154 differentially expressed pathways, of which the majority overlapped with pathways that were induced by the WTD (Figure 4A, Venn diagram, blue circle). Of this overlapping portion of the Venn diagram, the top 15 most significantly enriched pathways that were induced by the WTD but reversed with atorvastatin were mainly involved in lipid metabolism, but also acute phase response signaling and hepatic fibrosis/hepatic stellate cell activation (Figure 4A). The top 15 differentially expressed pathways solely regulated by atorvastatin reveal a diverse list that includes pathways involved in lipid metabolism, mitochondrial pathways and several different degradation pathways (Figure 4A).

Additionally, we analyzed upstream regulators by determining the activation state of transcription factors/master regulators, based on changes in the expression of their target genes. Activation states are expressed as z-scores and all significant upstream regulators with a z-score < −3.5 and >4.0 (arbitrary cut-offs to shorten the list) for WTD vs. chow and for WTD + atorvastatin vs. WTD are shown in Figure 4B. Since statins intervene in the cholesterol synthesis pathway by inhibiting HMG-CoA reductase, it was to be expected that atorvastatin exerted positive effects on the regulators of lipid metabolism. Furthermore, in line with our findings on hepatic inflammation, the transcriptomics data confirm that atorvastatin also strongly down-regulates pro-inflammatory upstream regulators. For example, WTD-induced upregulation of *MYD88* gene expression—a gene that decodes the MyD88 protein involved in NFκB signaling—was prevented by atorvastatin, similarly to IL33, IL1B and Ige.

Because of the beneficial effects of atorvastatin treatment on the components of the NLRP3 inflammasome pathway, we investigated this pathway more closely on the transcriptional level. Comparing the transcriptome profile of atorvastatin-treated mice to that of WTD-fed mice, we observed downregulation of Toll-like receptor 4 (TLR4), cathepsin B (CTSB) and nuclear factor kappa B (NFκB) (Figure 4C). Using Ingenuity Pathway Analysis to predict the upstream/downstream effects of activation/inhibition of NLRP3 inflammasome pathway components (based on the activity state of neighboring components), we found that atorvastatin was predicted to inhibit most of the genes in the NLRP3 inflammasome pathway that were induced by the WTD.

## 3. Discussion

Statins are often prescribed to hyperlipidemic patients to prevent the development or halt the progression of cardiovascular complications, while they are underprescribed in NAFLD-NASH patients, despite a large overlap in the clinical picture of the metabolic syndrome [24]. This underprescription is mostly due to safety concerns regarding liver function tests and hepatotoxicity, although comprehensive reviews have demonstrated that statins can be used safely for dyslipidemia management in these patients [24,25,26]. In the current study, we used APOE*3-Leiden mice to demonstrate that atorvastatin, one of the most-often prescribed statins in clinical practice [27], protects against development of diet-induced NASH, mainly by reducing hepatic inflammation and fibrosis. Furthermore, atorvastatin greatly reduced hepatic cholesterol levels, thereby protecting against the formation of hepatic cholesterol crystals—structures that promote inflammation by functioning as NLRP3 inflammasome ligands [21,23]. Analysis of hepatic gene expression further supported the effect of atorvastatin on regulatory pathways and upstream regulators involved in inflammation.

There is an emerging interest in how statins influence the development and progression of NAFLD-NASH, as is reflected by the rising number of clinical studies that have been done or are being carried out. Cholesterol-lowering interventions currently do not belong to the standard treatment approach, while emerging evidence advocates their beneficial effects in the context of NAFLD-NASH. Recent systematic reviews and meta-analyses demonstrate that in NAFLD-NASH patient populations, statins not only significantly improve total cholesterol levels [28] but also liver enzymes including ALT and AST [25,26,28]. Nevertheless, few prospective studies on this subject have been carried out and most have a short follow-up time (maximally one year), small study populations, and lack histologically confirmed diagnoses of the disease [8,29,30]. NAFLD-NASH is often diagnosed through imaging methods such as ultrasonography and magnetic resonance imaging, and while these methods are non-invasive and rapidly evolving, they are the less sensitive methods for the stratification between NAFLD and NASH [31]. The gold standard, hepatic biopsy, is an invasive method and accordingly, it is thought that the reported prevalence of NAFLD-NASH highly underestimates its true prevalence. Meta-analyses that looked in more detail at the effects of statins on hepatic complications in biopsy-confirmed NAFLD-NASH patients reported a significant improvement of their NAFLD activity score [25,28], which is a measure of steatosis, hepatocellular ballooning and lobular inflammation [32]. Improvement in the necro-inflammatory grade was also reported in a study on the effects of atorvastatin in 22 NASH patients [29] and a meta-analysis revealed significant reductions in necro-inflammation with statin intervention in a total of 40 NASH patients [25]. On the other hand, a prospective study in 101 biopsy-proven NASH patients did not report any ameliorative effects of statin intervention on liver histology, including steatosis, necro-inflammation or fibrosis after 18 months [33]. These large discrepancies in clinical trial data call for a more mechanistic approach to unravel the effects of cholesterol-lowering therapies like statins on the development and treatment of NAFLD-NASH.

Preclinical models are of great value in elucidating the molecular characteristics of NASH and fibrosis development. However, development of therapeutic interventions for the treatment of NAFLD-NASH has been hampered because these models do not respond to hyperlipidemic drugs with a lowering of plasma lipid concentrations, as seen in human NASH patients [10,11,13,34]. This makes these models less suitable for the evaluation of lipid-lowering interventions and their effects on NASH and fibrosis development. Therefore, we chose the APOE*3-Leiden mouse, a model that responds well to lipid-lowering therapeutics used in the clinic, including statins, with respect to their effects on plasma cholesterol and triglyceride levels [5,10,14,35], for example. In the current study, we used female WTD-fed APOE*3-Leiden mice, since they develop NASH and liver fibrosis [15,16,36] concomitantly with atherosclerosis [16,36,37] and respond well to statins [5,14,38]. However, each animal model has its limitations and for this particular model–diet combination, it is important to realize that the mice do not become obese, and the developed steatosis is primarily microvesicular steatosis resulting from increased hepatic cholesteryl ester concentrations, while their hepatic triglycerides were not elevated. Similarly, these mice do not develop insulin resistance [12], a characteristic that is observed in a subgroup of patients with metabolic syndrome. For a more translational obese and steatotic NASH model male, APOE*3-Leiden or APOE*3-Leiden.CETP mice fed a high fat and cholesterol (HFC) diet would have been more suitable. However, these male mice on HFC diets are less responsive to statins and less suitable to studying atherosclerosis development. Other preclinical model-diet combinations can be of great value for the investigation of pathogenic processes of NAFLD-NASH, yet are often less suitable for the evaluation of lipid-lowering interventions. Most of these models, including, for example, C57BL/6J mice, do not respond to statins in a similar way as most patients, with regard to the lowering of plasma cholesterol and triglycerides, which is the primary goal of statin intervention [39,40,41]. Due to the more human-like lipoprotein metabolism of the APOE*3-Leiden mouse model, these mice responded to statins in a way that corresponds to the large patient population, with a lowering of plasma cholesterol and triglyceride concentrations. The dosage used here is translational to the dosage used in human NASH patients and resulted in an average 30% reduction of total cholesterol levels, which is representative for a large group of patients in the mild-hyperlipidemic population [20]. To translate the dosage used in this study to human dosing, the following simplified calculation based on body surface area, as accepted by the FDA, can be used as a guide: (mouse dose/12.3)×human body weight [42]. Accordingly, 3.0–4.5 mg/kg/day of atorvastatin in mice corresponds to 20–30 mg/day for an 80-kg human. The effects of atorvastatin in this study were therefore achieved at a moderate dosage that was not hepatotoxic and was comparable to that used in the clinical setting.

The efficacy of statins in reducing atherosclerosis has been extensively described in the literature and has been confirmed in the WTD-fed APOE*3-Leiden mouse model [5,10,14,16]. In the present study, these ameliorative effects could mainly be attributed to the prevented formation of large and complex atherosclerotic lesions and increased overall plaque stability. At the hepatic level, the WTD-induced hypercholesterolemia resulted in increased liver weight, increases in plasma levels of liver damage marker ALT and AST, and increases in the plasma levels of the hepatic inflammation biomarker SAA, which all suggested liver damage. These parameters were all improved when the WTD was supplemented with atorvastatin, shifting liver weight, ALT, AST and SAA plasma concentrations towards levels observed in healthy, chow-fed mice. As previously indicated, improvement of plasma ALT and AST concentrations were in line with recent systematic reviews on the efficacy of statins for the treatment of NAFLD and NASH [25,26,28]. In the current study in APOE*3-Leiden mice, microscopic analysis revealed the positive effects of atorvastatin treatment on hepatic steatosis and inflammation. The improvement of hepatic fibrosis and fibrosis stage underline the potential of atorvastatin as a therapeutic agent for the management of NASH. Although the antisteatotic and antifibrotic effects of statins have been suggested before in other preclinical studies [43,44], we demonstrated the hepatoprotective potential of atorvastatin in a model that specifically develops NASH in conjunction with atherosclerosis and therefore shows high translatability to a broad population of NAFLD-NASH patients. In addition, the effects of statins on hepatic inflammation have not been studied in as much detail in the literature, and available data from clinical studies on the effects of statins on hepatic parameters in patients with biopsy-confirmed NAFLD-NASH are conflicting [29,33,45].

Further investigation concerning the effects of atorvastatin on inflammatory parameters revealed a reduction in the number of infiltrated macrophages and neutrophils, indicating that WTD-induced inflammation can be counteracted by atorvastatin. Moreover, since hepatic cholesterol concentrations exceeded physiological ranges in WTD-fed mice, free cholesterol accumulated in the form of crystals in hepatic cell membranes. Formation of hepatic cholesterol crystals is a phenomenon that is seen in patients with NASH, but not in patients with simple steatosis [46]. From a mechanistic perspective, in addition to the subcellular damage that cholesterol crystals may do by disrupting the integrity of intracellular structures, they are also recognized as triggers for inflammation via activation of the NLRP3 inflammasome [21,46]. As a result, cholesterol crystals stimulate production and maturation of the pro-inflammatory cytokines IL-1β and IL-18, thereby driving the progression from NAFLD into NASH [21,23,47]. A study that used human peripheral blood mononuclear cells from hyperlipidemic patients revealed a sharp rise in IL-1β activation upon stimulation with cholesterol crystals [48]. After these patients received statin treatment for eight weeks and the experiment was repeated, the response to cholesterol crystals was significantly lower than at baseline. Nevertheless, controversy on how statins affect NLRP3 inflammasome activation remains, since other papers propose that statins may promote IL-1β cleavage and activation [49], which is an effect that is mainly reported in studies that used simvastatin. Several studies suggest that the efficacy of statins on NLRP3 inflammasome activity may highly depend on the type of statin that is being used [50,51], which might be explained by differences in lipophilicity [52]. Little is known about the effects of statins on the NLRP3 inflammasome pathway activation in the context of NAFLD-NASH, and here we found that atorvastatin can limit activation of this pathway in the liver. Accordingly, hepatic protein levels of the effector pro-inflammatory cytokines IL-1β and IL-18 were significantly reduced in atorvastatin-treated mice. While we observed a 61% reduction in IL-1β levels, IL-18 levels were lowered by 26%, a difference that can be attributed to different regulatory mechanisms [53]. We infer that through its cholesterol-lowering properties, atorvastatin can prevent the formation of cholesterol crystals that serve as ligands for the NLRP3 inflammasome pathway and thereby can limit hepatic inflammation.

Further analysis revealed substantial changes in the hepatic lipidomics profile induced by WTD-feeding. A different study by Ooi et al. showed differences in the lipidomics profile of bariatric patients without NAFLD-NASH and with either NAFLD or NASH, and reported considerable changes in hepatic levels of various lipid species with increasing NAFLD-NASH severity [54]. Because no differences were found in the hepatic lipidomics profile of patients with NAFLD and patients with NASH, the authors suggest that changes in the hepatic lipidome likely present themselves in the early stages of NAFLD development. While a few studies have reported on changes in the hepatic lipid concentrations of healthy subjects when compared with NAFLD or NASH subjects, none of these studies make a clear distinction in which medication to use. Our data demonstrated that intervention with atorvastatin had significant normalizing effects on hepatic lipid concentrations. The APOE*3-Leiden mouse model on a WTD may be less suitable to evaluate the effects of atorvastatin on hepatic triglyceride content, since concentrations in chow-fed mice and WTD-fed mice were comparable (123.7 ± 19.3 in chow vs. 152.1 ± 11.3 nmol/g liver in WTD fed animals; which was not significant). At the transcriptional level, we discerned that atorvastatin downregulated the expression of genes involved in inflammatory pathways including TLR4, MyD88 and NFκB. The additional Ingenuity Pathway Analysis-predicted inhibition of other relevant genes in the NLRP3 inflammasome pathway by atorvastatin confirms the significance of atorvastatin in preventing an inflammatory response. Together, these data demonstrate in detail the beneficial effects of atorvastatin on hepatic lipid content as well as on hepatic inflammation.

In summary, we demonstrated that atorvastatin prevents development of hepatic steatosis, inflammation and fibrosis in APOE*3-Leiden mice, a model that develops atherosclerosis and NASH upon WTD-feeding. Additionally, we have shown that atorvastatin prevents the crystallization of free cholesterol in the liver, thereby averting activation of the NLRP3 inflammasome. All in all, the data in this study underline the anti-inflammatory properties of atorvastatin in the context of NASH and add to this knowledge by emphasizing its favorable effects on hepatic steatosis and fibrosis. Accordingly, atorvastatin intervention may be an attractive approach to reduce existing NAFLD and additional cardiovascular risk, and prevent progression into NASH in a broad population of dyslipidemic patients.

## 4. Materials and Methods

### 4.1. Animals and Experimental Design

The study was approved by the governmental central committee on animal experiments (AVD5010020172064) and the animal welfare body of The Netherlands Organization for Applied Scientific Research (IVD TNO; TNO-452/TNO-459) and was in compliance with European Community specifications regarding the use of laboratory animals. APOE*3-Leiden transgenic mice were housed and bred at the AAALAC-accredited SPF animal facility at TNO (TNO Leiden). Female animals were used for this study, since they develop NASH and liver fibrosis [15,16,36] concomitantly with atherosclerosis [16,36,37] and respond well to statins [5,14,38].

During this study, mice were group-housed in a temperature-controlled room (21 ± 2 °C) on a 12 h light-dark cycle at 50–60% humidity and had free access to heat-sterilized tap water and food. Body weight, food intake (per cage) and clinical signs were monitored regularly. The number of animals per group was calculated a priori with a power analysis using GPower3.1, based on a minimal effect size of 50%, standard deviation of 50% and using a one-sided test with a 95% confidence interval and power of 0.85. Female APOE*3-Leiden mice (8–14 weeks old) were fed a semi-synthetic Western-type diet (WTD) which contained, among other ingredients, 15% (*w*/*w*) cocoa butter, 40.5% (*w*/*w*) sucrose and 1% (*w*/*w*) cholesterol (Ssniff Spezialdiäten GmbH, Soest, Germany) for a run-in period of three weeks. Hereafter, at t = 0, mice were matched for age, body weight and 4 h-fasted plasma cholesterol and triglycerides into two groups of *n* = 16 each. From baseline (t = 0) onward, one of these two WTD-fed groups was provided 3.0 mg/kg/day atorvastatin (Lipitor pills, obtained from the Leiden University Medical Center pharmacy) as admixture to the diet. At t = 5 weeks, the atorvastatin dose was increased to 4.5 mg/kg body weight/day to obtain a 30% decrease in total cholesterol levels, as is representative for a large group of patients in the mild-hyperlipidemic population [20]. Atorvastatin was selected because of its broad dosing window and the absence of hepatotoxic effects within these dosages [14,19,35]. At t = 22 weeks, the WTD was supplemented with 0.05% (*w*/*w*) sodium cholate (Ssniff Spezialdiäten GmbH, Soest, Germany) to further raise plasma cholesterol levels. Mice continued on this diet for another ten weeks to further increase hepatic fibrosis and atherosclerosis development. At t = 32 weeks, animals were sacrificed by CO_2_ asphyxiation, terminal blood was collected through heart puncture, and livers, hearts and aortic roots were fixed in formalin or snap-frozen in liquid nitrogen. In addition to the two WTD-fed groups, a group of ten mice that were kept on a maintenance chow diet (Ssniff Spezialdiäten) was included as healthy reference.

### 4.2. Biochemical Analyses in Plasma and Liver Tissue

Every 2–3 weeks, blood samples for analysis of plasma parameters were collected from the tail vein into EDTA-coated tubes (Sardtedt, Nümbrecht, Germany) after a 4-h fasting period. Plasma total cholesterol (TC) and triglycerides (TG) were determined using enzymatic colorimetric assays (Roche Diagnostics, Almere, The Netherlands). Total cholesterol exposure was expressed as mM cholesterol multiplied by the duration of the study in weeks. Plasma aspartate transaminase (AST) and alanine transaminase (ALT) levels were determined by reflectance photometry using a Reflotron^®^ Plus analyzer (Hoffman-La Roche, Mannheim, Germany). Enzyme-linked immunosorbent assays (ELISAs) were used to determine plasma levels of serum amyloid A (SAA, #KMA0021; Thermo Fisher Scientific, Waltham, MA, USA) and hepatic levels of interleukin (IL)-1β (ab197742; Abcam, Cambridge, UK) and IL-18 (ab216165; Abcam). Hereto, liver tissue samples were homogenized in lysis buffer containing Tris-HCl pH 7.4, 150 mmol/L NaCl, 5 mmol/L CaCl_2_ (*v*/*v*), 1% Triton X-100 and cOmplete^TM^ mini protease inhibitor cocktail (Roche Diagnostics) and centrifuged for 30 min at 4 °C, 16,200× *g*, after which the supernatant was used for analysis. All procedures were carried out in accordance with the manufacturer’s protocols. For hepatic cholesterol analysis, liver tissue samples were homogenized in phosphate-buffered saline and lipids were extracted and measured as described previously [55,56].

### 4.3. Histological Assessment of NASH

Livers were fixed in 4% paraformaldehyde, embedded in paraffin and cross-sectioned (3 µm). Sections were stained with hematoxylin-eosin (H&E) and Sirius Red (SR) and scored blindly by a board-certified pathologist for NASH. In short, steatosis and inflammation were scored in two H&E-stained liver slides per mouse using an adapted grading system of human NASH [32,57]. Steatosis was determined by analyzing hepatocellular vacuolization and hepatocellular hypertrophy relative to the total liver area analyzed. Inflammation was scored by counting the number of aggregates of inflammatory cells per field using a 100× magnification (view size of 4.2 mm^2^). The averages of five random non-overlapping fields were taken and values were expressed per mm^2^. The development of hepatic fibrosis was assessed blindly by a board-certified pathologist using SR-stained slides to quantify the percentage of perisinusoidal fibrosis (expressed as the percentage of perisinusoidal fibrosis relative to the total perisinusoidal area). The stage of hepatic fibrosis was evaluated using an adapted scoring protocol by Tiniakos et al. [58], where F0 indicates absence of fibrosis, F1 fibrosis observed in perisinusoidal/perivenular or periportal area, F2 fibrosis within both perisinusoidal and periportal areas, F3 bridging fibrosis and F4 cirrhosis. To evaluate the presence of birefringent cholesterol crystals, frozen liver sections were formalin-fixed and stained with H&E and examined by bright-field microscopy with or without a polarizing filter as described previously [15].

### 4.4. Histological Assessment of Atherosclerosis

A histological assessment of atherosclerosis was done as previously described [59,60]. In short, formalin-fixed aortic roots were embedded in paraffin and serial cross-sections of the entire aortic root area (5 µm thick with 50 µm intervals) were mounted on slides and stained with hematoxylin-phloxine-saffron (HPS). Per mouse, four sections at 50 µm intervals were used for the assessment of atherosclerotic lesions using an Aperio AT2 slide scanner (Leica Biosystems, Amsterdam, The Netherlands). Lesions were classified into five categories according to the American Heart Association classification: (I) early fatty streak, (II) regular fatty streak, (III) mild plaque, (IV) moderate plaque, and (V) severe plaque [19,60]. Smooth muscle cell (SMC) and macrophage content of type IV and V lesions was assessed by ImageJ software (version 1.48; NIH) and customized macros after double immunostaining with anti-α-smooth muscle actin (PROGEN Biotechnik GmbH, Heidelberg, Germany) for SMCs, and anti-mouse LAMP2 (M3/84) (MA5-17861; Invitrogen, Waltham, MA, USA) for macrophages. Anti-α-smooth muscle actin was labeled with Vina green (Biocare Medical, Pacheco, CA, USA) and LAMP2 with DAB (Vector Laboratories, Burlingame, CA, USA). After slides were scanned and analyzed, cover slips were detached overnight in xylene and an SR staining for collagen was performed. Collagen content and necrotic core content (including cholesterol clefts) were analyzed using ImageJ in SR-stained slides. Plaque stability index, calculated by dividing the sum of SMC and collagen content (i.e., as stabilizing factors) by the sum of macrophage and necrotic core content (i.e., as destabilizing factors) was calculated as described previously [16,19,35].

### 4.5. Immunohistochemistry

Paraffin-embedded hepatic cross-sections (3 µm) were used for immunohistochemical analysis of markers of inflammation. Sections were deparaffinized in xylene, followed by endogenous peroxidase blockage with 0.03% H_2_O_2_ in methanol for 20 min and subsequent rehydration in graded concentrations of ethanol/water. Epitope retrieval was performed at 97 °C for 20 min at pH 6.0 by using the Dako PT Link (Agilent Technologies, Amstelveen, The Netherlands), followed by blocking in a buffer containing 5% normal goat serum (NGS) in PBS. Primary antibody incubation in 1% normal goat serum in PBS took place overnight at 4 °C, after which the sections were washed with 0.05% (*v*/*v*) Tween-20 in PBS and incubated with the horseradish peroxidase (HRP)-conjugated secondary antibodies in 1% (*v*/*v*) NGS in PBS for 1 h at room temperature. HRP activity was visualized using 3,3′-diaminobenzidine (DAB; Vector Laboratories), after which slides were dehydrated, counterstained with hematoxylin (Sigma-Aldrich, St. Louis, MO, USA) and mounted using a Leica CV5030 Fully Automated Glass Coverslipper (Leica Biosystems, Amsterdam, The Netherlands). Imaging was done with the Aperio AT2 imaging system (Leica Biosystems) at 20× magnification. For CD68 staining, a rabbit polyclonal anti-mouse antibody (ab125212-1:200 *v*/*v*; Abcam, Cambridge, UK) and HRP-conjugated goat anti-rabbit secondary antibody (ab97080-1:400 *v*/*v*; Abcam) were used. The same secondary antibody was used for visualization of myeloperoxidase (MPO, ab9535-1:100 *v*/*v*; Abcam). For immunohistochemical staining of Ly-6C and Ly-6G, a monoclonal rat anti-mouse antibody (#550291-1:100 *v/v;* BD Biosciences, Vianen, The Netherlands) was combined with an HRP-conjugated goat anti-rat secondary antibody (ab97057, 1:800 *v/v;* Abcam). For staining of hepatic NLRP3, a rabbit polyclonal anti-mouse antibody (#PA-79740-1:200, *v*/*v*, Invitrogen, Waltham, MA, USA) and HRP-conjugated goat anti-rabbit secondary antibody (ab205718-1:1000; Abcam) were used. Quantification was performed in ImageJ (version 1.48, NIH) with customized macros, for which two cross-sections per mouse were evaluated at 20× magnification and the positively stained area was determined in five non-overlapping fields (view size 1.0 mm^2^).

### 4.6. Hepatic Lipidomic Analysis

Hepatic lipidomic analysis in snap-frozen liver tissue was performed as previously described in detail [61,62]. Briefly, the commercial Lipidyzer platform was used, which employs differential mobility spectrometry (DMS) on a Sciex 5500 QTrap mass spectrometer that is equipped with SelexIon technology. The Shotgun Lipidomics Assistant (SLA), an open-source application that analyzes DMS lipidomics data to generate quantitative results, was used to perform data normalization and concentrations were expressed as nmol/mg liver.

### 4.7. Gene Expression and Pathway Analysis

Transcriptomics analysis by Next Generation Sequencing was performed as described previously [63]. In short, total RNA was isolated from snap-frozen liver tissue using the RNA-Bee total-RNA isolation kit (Bio-Connect, Huissen, The Netherlands) and was purified with NucleoSpin RNA Clean-up (Macherey-Nagel, Düren, Germany). RNA concentrations were determined by Nanodrop 1000 (Isogen Life Science, De Meern, The Netherlands) and RNA quality was evaluated with 2100 Bioanalyzer (Agilent Technologies). Isolated RNA was used for generation of strand-specific mRNA-seq libraries for next-generation sequencing at GenomeScan B.V. (Leiden, The Netherlands). The NEBNext Ultra II Directional RNA Library Prep Kit (NEB #E7760S/L, New England Biolabs, Ipswich, MA, USA) was used to process the samples. Briefly, mRNA was isolated from total RNA using the oligo-dT magnetic beads. After fragmentation of the mRNA, cDNA synthesis was performed and ligated with the sequencing adapters and amplified by PCR. Quality and yield of the amplicon was measured (Fragment Analyzer, Agilent Technologies) and was as expected (broad peak between 300–500 bp), and a concentration of 1.1 nM of amplicon-library DNA was used. Clustering and DNA sequencing using the Illumina NovaSeq6000, was performed according to the manufacturer’s protocols of the service provider GenomeScan B.V., yielding 20–40 million sequencing clusters per sample and 2 × 150 bp Paired-End reads (PE) per cluster. The genome reference and annotation file Mus_musculus.GRCm38.gencode.vM19 was used for analysis. The reads were aligned to the reference sequence using the STAR 2.5.3a algorithm accessed on 17 March 2017 with default settings (https://github.com/alexdobin/STAR). Based on the mapped read locations and the gene annotation, HTSeq-count version 0.6.1p1 was used to count how often a read is mapped on the transcript region. These counts served as input for the statistical analysis using DEseq2 package 4. Selected differentially expressed genes (DEGs), corrected for multiple testing, were used as an input for pathway analysis (Padj < 0.01) through Ingenuity Pathway Analysis suite (IPA, Ingenuity Systems Inc., Redwood City, CA, USA, www.ingenuity.com, accessed February 2022) to identify differentially expressed pathways and upstream regulators. IPA determines the activation state of transcription factors based on the observed differential gene expression where *z*-scores greater than 2 indicate enhanced activity and *z*-scores less than −2 indicate reduced activity of upstream regulators [63]. The *p*-values indicate the significance of gene enrichment of these upstream regulators. The gene expression dataset of this study is accessible via Gene Expression Omnibus (GEO), accession number GSE229188.

### 4.8. Statistical Analysis

Data are presented as mean ± standard error of the mean (SEM). Differences between groups were determined by means of non-parametric Kruskal–Wallis testing, followed by Mann–Whitney U testing for independent samples. SPSS software (version 25; IBM Corp., Armonk, NY, USA) was used and a *p*-value < 0.05 was considered statistically significant. Two-tailed *p*-values were used. IPA analysis to determine differentially expressed genes were based on Fisher’s exact test (*p* = 0.01).

## Figures and Tables

**Table 1:** Metabolic risk factors at the study endpoint.

	Chow	WTD	WTD + Atorvastatin
Body weight (g)	25.9 ± 0.8	25.9 ± 0.3	25.5 ± 0.5
Food intake (kCal/mouse/day)	12.7 ± 0.2 ***	11.3 ± 0.2	11.4 ± 0.1
Plasma cholesterol (mM)	2.4 ± 0.1 ***	27.8 ± 1.6	15.8 ± 0.7 ***
Plasma triglycerides (mM)	2.6 ± 0.2 **	5.0 ± 0.4	2.8 ± 0.2 ***
Liver weight (g)	1.3 ± 0.1 ***	2.5 ± 0.1	1.8 ± 0.1 ***
Plasma alanine transaminase (U/L)	50.8 ± 6.0 ***	283.2 ± 21.1	143.8 ± 16.5 ***
Plasma aspartate transaminase (U/L)	110.2 ± 14.9 ***	782.8 ± 48.2	521.2 ± 59.0 **
Plasma serum amyloid A (µg/mL)	11.6 ± 0.4 ***	23.6 ± 1.6	13.7 ± 0.9 ***

Data were determined at the study endpoint (t = 32 weeks) and are presented as mean ± SEM for *n* = 10 mice on chow, *n* = 16 mice on Western type diet (WTD) and *n* = 16 mice on WTD supplemented with atorvastatin. ** *p* < 0.01, *** *p* < 0.001 vs. WTD.

## Data Availability

The data presented in this study are available in this article and the accompanying Supplementary Material. The mouse gene expression dataset used for transcriptomics analysis is publicly available via Gene Expression Omnibus, accession number GSE229188.

## Supplementary Materials

The supporting information can be downloaded at: https://www.mdpi.com/article/10.3390/ijms24097818/s1.
