# Supplementary material for: Atorvastatin Attenuates Diet-Induced Non-Alcoholic Steatohepatitis in APOE*3-Leiden Mice by Reducing Hepatic Inflammation
Source: Int J Mol Sci. 2023 Apr 25;24(9):7818. doi: 10.3390/ijms24097818 (PMC10178767; doi:10.3390/ijms24097818)
Supplement: Supplementary file 1 [file ijms-24-07818-s001.zip › ijms-2348358-supplementary.pdf]

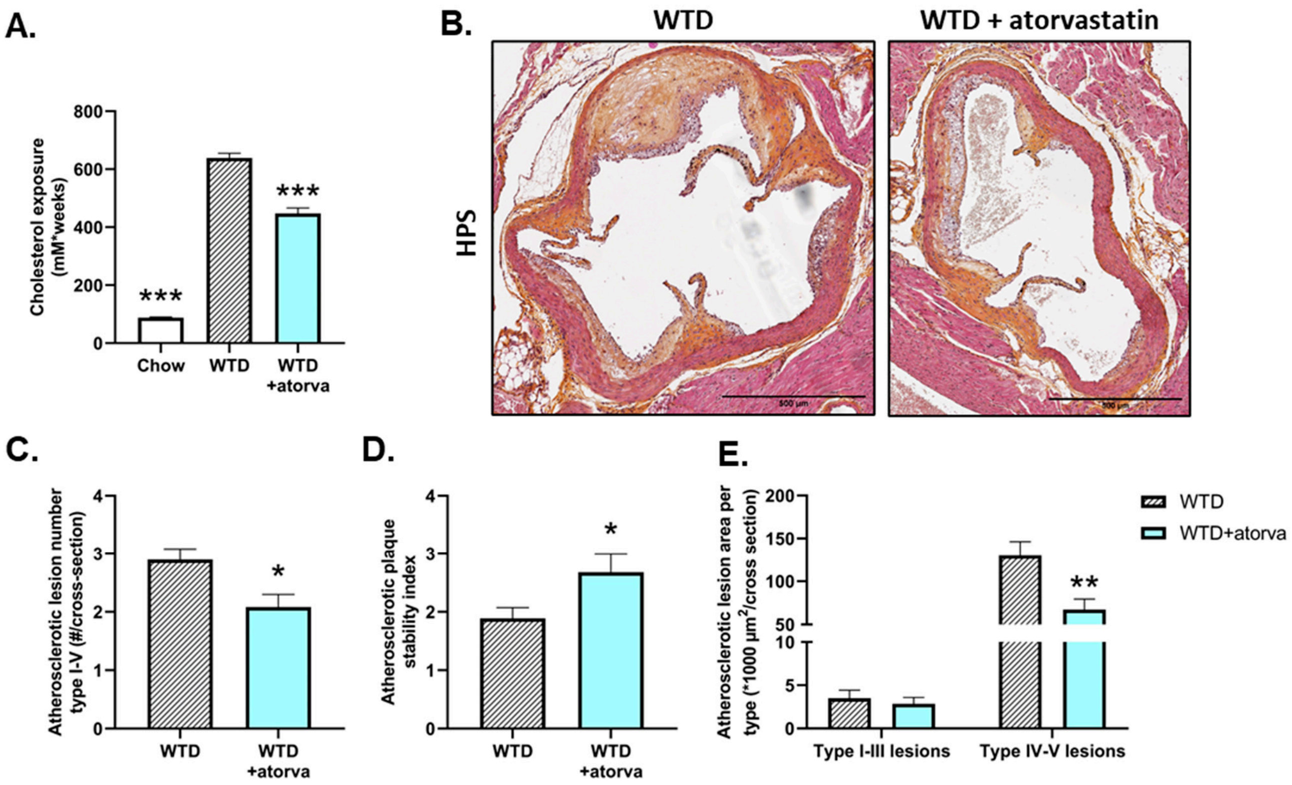

Figure S1. Atherosclerosis parameters improve with atorvastatin treatment. APOE*3-Leiden mice were fed a Western type diet (WTD) with (n=16) or without (n=16) atorvastatin admix for 32 weeks. Chow-fed mice (n=10) were included as healthy reference for cholesterol exposure but do not develop atherosclerotic lesions. Cholesterol exposure (concentration*weeks) (**A**), representative histological photomicrographs of HPS-stained cross-sections of the aortic root area (**B**), number of type I-V atherosclerotic lesions (**C**), atherosclerotic plaque stability index (smooth muscle cell + collagen content divided by macrophage + necrotic core content) (**D**) and atherosclerotic lesion area per lesion type (**E**) were determined at the study endpoint (t=32 weeks) and are presented as mean ± SEM *p<0.05, **p<0.01, ***p<0.001 vs. WTD.
